# Taurine Treatment Modulates Circadian Rhythms in Mice Fed A High Fat Diet

**DOI:** 10.1038/srep36801

**Published:** 2016-11-18

**Authors:** Ana Lucia C. Figueroa, Hugo Figueiredo, Sandra A. Rebuffat, Elaine Vieira, Ramon Gomis

**Affiliations:** 1 Diabetes and Obesity Research Laboratory, Institut d’Investigacions Biomèdiques August Pi i Sunyer (IDIBAPS), Barcelona, Spain; 2 CIBER de Diabetes y Enfermedades Metabólicas Asociadas (CIBERDEM), Spain; 3 Postgraduate Program on Physical Education, Universidade Católica de Brasília-UCB, DF, Brazil; 4 University of Barcelona, Faculty of Medicine, University of Barcelona, Barcelona, Spain.

## Abstract

Close ties have been made among certain nutrients, obesity, type 2 diabetes and circadian clocks. Among nutrients, taurine has been documented as being effective against obesity and type 2 diabetes. However, the impact of taurine on circadian clocks has not been elucidated. We investigated whether taurine can modulate or correct disturbances in daily rhythms caused by a high-fat diet in mice. Male C57BL/6 mice were divided in four groups: control (C), control + taurine (C+T), high-fat diet (HFD) and HFD + taurine (HFD+T). They were administered 2% taurine in their drinking water for 10 weeks. Mice were euthanized at 6:00, 12:00, 18:00, and 24:00. HFD mice increased body weight, visceral fat and food intake, as well as higher levels of glucose, insulin and leptin, throughout the 24 h. Taurine prevented increments in food intake, body weight and visceral fat, improved glucose tolerance and insulin sensitivity and reduced disturbances in the 24 h patterns of plasma insulin and leptin. HFD downregulated the expression of clock genes *Rev-erbα*, *Bmal1*, and *Per1* in pancreatic islets. Taurine normalized the gene and protein expression of PER1 in beta-cells, which suggests that it could be beneficial for the correction of daily rhythms and the amelioration of obesity and diabetes.

Diverse physiological and behavioral circadian oscillations such as sleep-wake cycles and the secretion of hormones and metabolism are controlled by the molecular clock that generates daily rhythms in mammals. This clock allows for adaptation to periodic changes in the environment, according to light and dark cycles^1^,^2^.

Circadian rhythms are controlled by cell-autonomous and self-sustained oscillators, which depend on the transcription-translation, autoregulatory feedback loop of specific clock genes. The positive limb is formed by transcription factors, including *Bmal1/Clock*, that activates the transcription of *Per and Cry* genes, which drive the negative limb and inhibit the activity of *Bmal1/Clock*, generating rhythmic oscillations of gene expression. An additional regulation is driven by the nuclear receptors *Reverb’s* and *Ror’s*, which repress and activate *Bmal1*, respectively^3^,^4^,^5^. Mammalian circadian clocks are composed of a central clock located in the suprachiasmatic nucleus (SCN) in the hypothalamus and several peripheral clocks found in many tissues; including the pancreas, the liver, adipose tissue and muscle^6^,^7^,^8^,^9^,^10^,^11^,^12^. Peripheral clocks contribute to global energy metabolism and participate in local metabolic functions, such as those involved in glucose and lipid homeostasis. Among peripheral clocks, the pancreatic clock is crucial for controlling the function of pancreatic beta-cells. Ablation of *Clock* and *Bmal1* with genetic manipulation led to the development of diabetes in mice, whereas *Rev-erbα* gene silencing led to impairments in beta- and alpha-cell function^8^,^10^,^11^,^13^.

Other factors apart from genetic manipulation can also disrupt clock gene expression in peripheral tissues. For instance, nutritional insults such as HFD feeding disrupted the expression of *Rev-erbα*, *Clock*, *Per1*, and *Per2* in mouse pancreatic islets, followed by disruption of the pattern of insulin secretion^10^. In addition, nutrient signaling, by glucose or amino acids may act as a training agent of SCN and peripheral clocks, leading to tissue-specific differences in the expression of clock genes^14^,^15^,^16^. Dietary supplementation of the amino sulfonic acid taurine was found to improve whole body glucose control particularly, and to be useful in both prevention and treatment for metabolic complications in obese rodents^17^,^18^. In this point, taurine is considered an essential nutrient due to the diverse therapeutic effects at different levels and could be proposed as a supplement in obesity treatment. Otherwise important external stimuli, like nutrients, synchronize the circadian clock, however the effect of taurine on daily rhythms of hormones and on the expression of clock genes has never been studied before. Therefore, the aim of this study was to determine whether taurine treatment can modulate and prevent disturbances of daily rhythms of hormones and the expression of clock genes caused by HFD.

## Results

### Effects of taurine treatment on body weight, visceral fat and food intake

**Figure 1:**
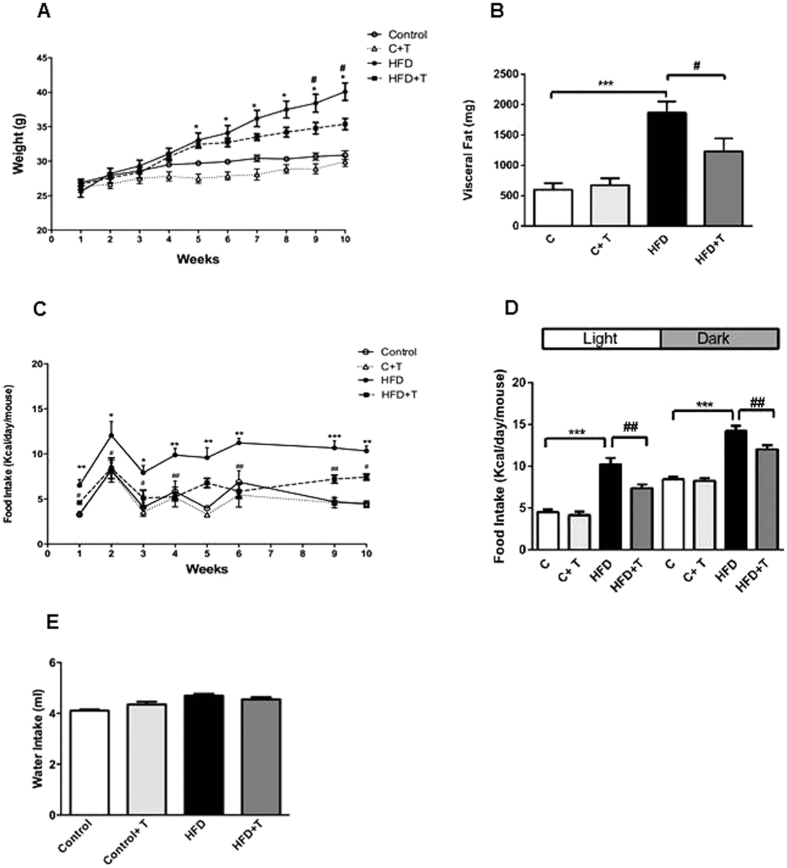
Effects of taurine treatment on body weight, visceral fat, food intake. Mice were treated with chow diet 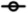 (C), Chow + Taurine 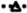 (C + T), High-fat diet 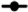 (HFD) or HFD + Taurine 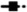 (HFD + T) for 10 weeks. (**A**) Body weight progression from the first day of taurine treatment until the 10^th^ week of treatment in mice fed with chow or HFD. (n = 8–10 mice per group). Differences between C vs HFD (*P < 0.05); HFD vs HFD+T (^**#**^P < 0.05, ^**##**^P < 0.01). (**B**) Visceral fat weight after 10 weeks of taurine treatment in mice fed with chow or HFD. Differences between C vs HFD (*P < 0.05); HFD vs HFD+T (^**#**^P < 0.05). (n = 8–10 mice per group). (**C**) Food intake progression from the first week until the 10th week of treatment. Differences between C vs HFD *P < 0.05. **P < 0.001, ***P < 0.0001. Differences between HFD vs HFD+T (^#^P < 0.05, ^##^P < 0.001). (**D**) Food intake at 10^th^ week of treatment during the light and dark cycle. Food was measured by weighing the food consumption separately during the day (from 8:00 to 20:00) and the food consumption during the night (from 20:00 to 8:00). Differences between C vs HFD (***P < 0.001); and HFD vs HFD+T (^**##**^P < 0.01). (**E**) Water Intake at 10^th^ week of treatment. Data are expressed as mean ± SEM.

We first measured body weight progression from the first week of treatment. Body weight was similar between the groups until the 5^th^ week of treatment. In the HFD group, body weight increased from the 5^th^ week of treatment in both HFD and HFD+T treated mice, as compared to controls. However, from the 8^th^ to the 10^th^ week of treatment, mice fed with HFD+T prevented the increase in body weight, compared to mice fed with HFD until the end of treatment (Fig. 1A). After 10 weeks of treatment, visceral fat weight was comparable between the C and C+T groups. As expected, mice treated with a HFD showed a significant increase in visceral fat, compared to the C group, whereas mice treated with HFD+T had a decrease in visceral fat, compared to the HFD group (Fig. 1B). Interestingly, HFD-treated mice had increased food intake already at the first week of treatment with a peak of food consumption at the second week and a sustained elevation of food intake until the end of treatment. On the other hand, HFD+T mice had decreased food intake already at the first week compared to the HFD group (Fig.1C). Measurements of food intake at the end of treatment (10^th^ week) during the light cycle was similar between C and C+T groups and increased during the dark cycle in both groups (P < 0.0001, respectively). In contrast, mice fed a HFD exhibited an increase in food intake during both light and dark cycles, as compared to C group (Fig. 1D). Strikingly, taurine decreased food intake in both light and dark cycles. Water intake was similar in all experimental groups at the 10^th^ week of treatment (Fig. 1E). The effects of the HFD and taurine treatment after 10 weeks on body weight gain, visceral fat and food intake and their interactions are also shown in Table 1. There was no effect of taurine treatment on body weight, visceral fat and food intake in the control group (Table 1). However, the effect of HFD alone when compared to the control group showed that this diet increased body weight, visceral fat and food intake. On the other hand, taurine treatment in the HFD group was able to decrease body weight, visceral fat and food intake although the treatment could not decrease these parameters at the level of the control group (Table 1). To check the effect of taurine at the beginning of the treatment we next measured body weight, visceral fat, water and food intake at the first week of treatment. There was no difference in body weight, visceral fat and water intake in all experimental groups (Supplementary Fig. 1A–C, respectively). Strikingly, mice fed a HFD had an increase in food intake during the light and dark cycles already at the first week of treatment whereas taurine was able to prevent the increase in food intake caused by HFD in both light and dark cycles (Supplementary Fig. 1D). Moreover, one week of HFD treatment led to glucose intolerance (Supplementary Fig. 2A) and decreased insulin sensitivity (Supplementary Fig. 2C) but taurine treatment in mice fed a HFD had a small effect on glucose tolerance and insulin sensitivity with no statistically significant results when calculating the area under the curve (Supplementary Fig. 2B,D respectively). These results showed that one week of taurine treatment in mice treated with HFD can decrease food intake during light and dark cycles even before any change in body weight and visceral fat. In addition, prolonged taurine treatment can modulate the increase in body weight, visceral fat and food intake caused by HFD feeding.

### Effects of taurine treatment on daily glucose, insulin and leptin levels

**Figure 2:**
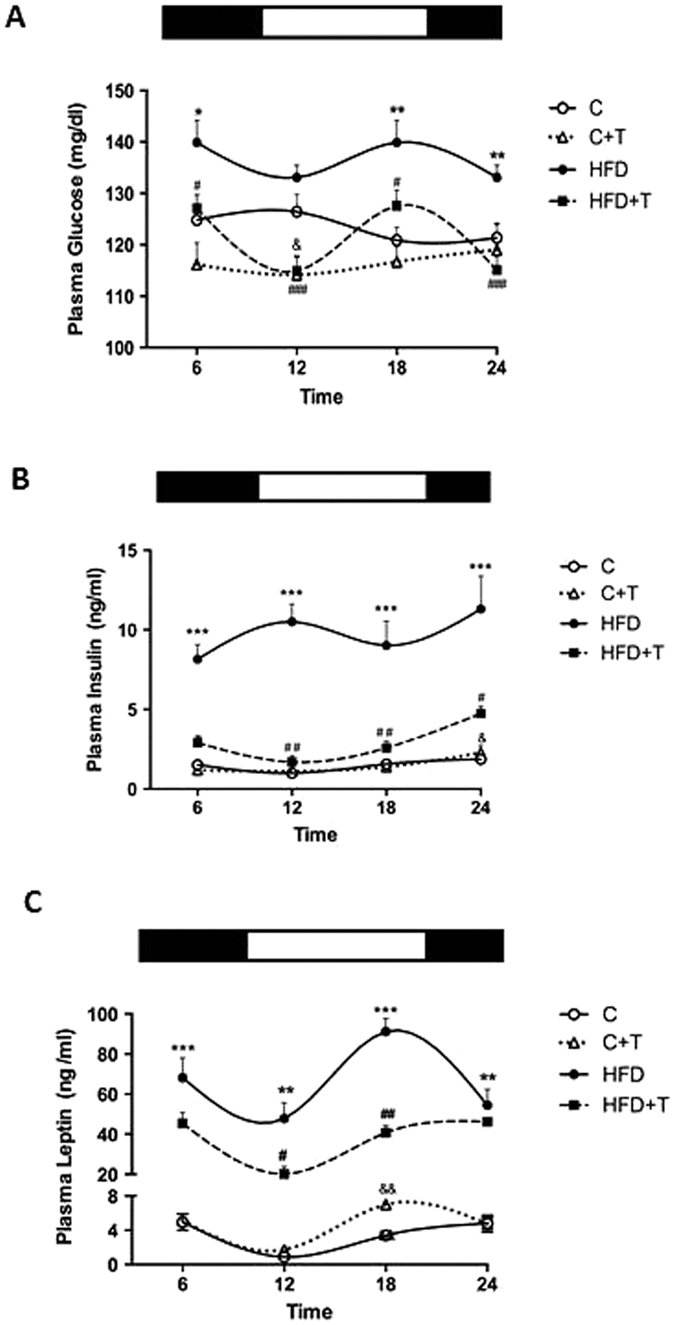
Effects of taurine treatment on daily glucose, insulin and leptin levels. (**A**) 24h time course measurements of plasma glucose concentrations after 10 weeks of taurine treatment in mice fed a chow diet or HFD. 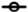 (C), 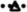 (C+T) 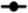 (HFD) and 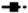 (HFD+T). Differences between C vs C+T (^&^P < 0.05). Differences between C vs HFD (*P < 0.05, **P < 0.001). Differences between HFD vs HFD+T (^#^P < 0.05 ^###^P < 0.001). (n = 8–10 mice per group). (**B**) 24 h time course measurements of plasma insulin concentrations after 10 weeks of taurine treatment in mice fed a chow diet or HFD. 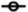 (C), 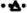 (C+T) 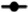 (HFD) and 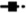 (HFD+T). Differences between C vs C+T (^&^P < 0.05). Differences between C vs HFD (***P < 0.001). Differences between HFD vs HFD+T (^#^P < 0.05, ^##^P < 0.01) (n = 8–10 mice per group). (**C**) 24 h time course measurements of plasma leptin concentrations after 10 weeks of taurine treatment in mice fed a chow diet or HFD 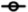 (C), 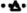 (C+T) 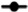 (HFD) and 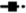 (HFD+T). Differences between C vs C+T (^&&^P < 0.01). Differences between C vs HFD (**P < 0.05, ***P < 0.001). Differences between HFD vs HFD+T (^#^P < 0.05, ^##^P < 0.01). (n = 8–10 mice per group). The black bars refers on the top of the figures to the dark cycle and the white bars to the light cycle.

The effect of taurine treatment (C+T group) on the daily glucose levels was evident at time 12:00, with lower levels than in the C group (Fig. 2A). As expected, blood glucose levels were elevated at 18:00 and 24:00 in the HFD group, compared to the C group (Fig. 2A). However, mice treated with HFD+T exhibited lower glucose levels throughout the 24 h period as compared to mice treated only with HFD (Fig. 2A). Mesor analysis of the data confirmed the hypoglycemic effect of taurine during HFD treatment by decreasing glucose levels to the same levels of the control group (Table 2).

Plasma insulin levels were similar in the C group at all time points measured, but increased in the C+T group at 24:00 (Fig. 2B). In contrast, insulin levels were continuously elevated throughout the 24 h period in the HFD group as compared to the C group. Interestingly, HFD+T decreased the overall levels of plasma insulin throughout the 24 h period and increased plasma insulin levels at 24:00 (Fig. 2B).

Control mice exhibited statistically significant variations in plasma leptin concentrations with decreased values at 12:00, and a peak at 24:00 (P < 0.01 and P < 0.05). The C+T group exhibited the same variations in plasma leptin, but the peak occurred at 18:00 (Fig. 2C). Interestingly, HFD mice disrupted the daily pattern of leptin, with no decrease in leptin levels at 12:00 and a peak of leptin at 18:00 (P < 0.05). Taurine treatment prevented the disruption of daily plasma leptin caused by HFD, decreased leptin levels at 12:00 (P < 0.01) and a peak of leptin from 12:00 to 24:00 (P < 0.01). Confirming these results, the mesor and amplitude values showed that taurine decreased insulin and leptin levels in mice treated with a HFD (Table 2). Our results demonstrated that taurine supplementation in mice fed a HFD can restore the 24 h pattern of plasma leptin levels.

### Effects of taurine on glucose tolerance and insulin sensitivity

**Figure 3:**
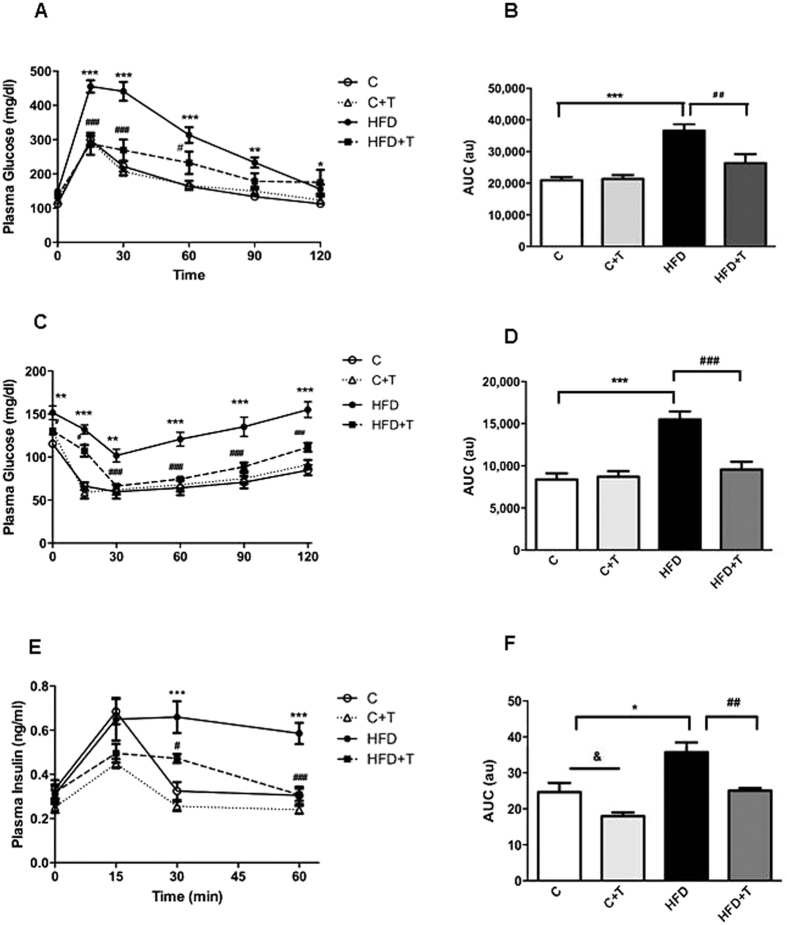
Effects of taurine treatment on glucose tolerance and insulin sensitivity. Glucose tolerance test (**A**) 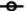 (C), 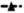 (C+T), 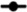 (HFD) and 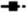 (HFD+T). Differences between C vs HFD (*P < 0.05, **P < 0.01 ***P < 0.001); HFD vs HFD+T (^#^P < 0.05, ^###^P < 0.001). (n = 8–10 mice per group). (**B**) Area under the curve (AUC). Differences between C vs HFD (***P < 0.001) and HFD vs HFD+T (**P < 0.01). (**C**) Insulin tolerance test (n = 8–10). Differences between C vs HFD (**P < 0.01 ***P < 0.001); HFD vs HFD+T (^#^P < 0.05, ^###^P < 0.001). (**D**) Area under the curve (AUC), (***P < 0.001). (**E**) Plasma insulin measured during the glucose tolerance test at 0, 15, 30, and 60 min (n = 5). Differences between C vs HFD (***P < 0.001); HFD vs HFD+T (^#^P < 0.05, ^###^P < 0.001) and C vs C+T (^&&^P < 0.01). (n = 8–10 mice per group).

Glucose tolerance was similar between C and C+T mice, showing no differences between the groups. As expected, HFD mice displayed impaired glucose tolerance, evident in the total area under the curve (AUC) as compared to control mice (Fig. 3A,B). Taurine supplementation in HFD-fed mice prevented glucose intolerance caused by a HFD, as indicated in the total area under the curve (AUC) (Fig. 3B). There were no differences in insulin sensitivity between C and C+T mice, but the HFD group exhibited impaired insulin sensitivity (Fig. 3C). Ten weeks of taurine treatment were sufficient to prevent insulin resistance caused by HFD feeding, as shown in the total area under the curve (AUC) (Fig. 3C,D). Plasma insulin levels during the ipGTT showed a significant decrease in plasma insulin levels in the C+T group at 15 min. In accordance with the ipGTT results, the HFD group displayed elevated insulin levels at 30 and 60 min, as compared to the C group. Interestingly, taurine treatment reduced insulin levels at 30 min and 60 min in animals treated with HFD (Fig. 3E), as indicated in the total area under the curve (AUC) (Fig. 3F).

### Effects of taurine on daily insulin secretion *in vitro*

**Figure 4:**
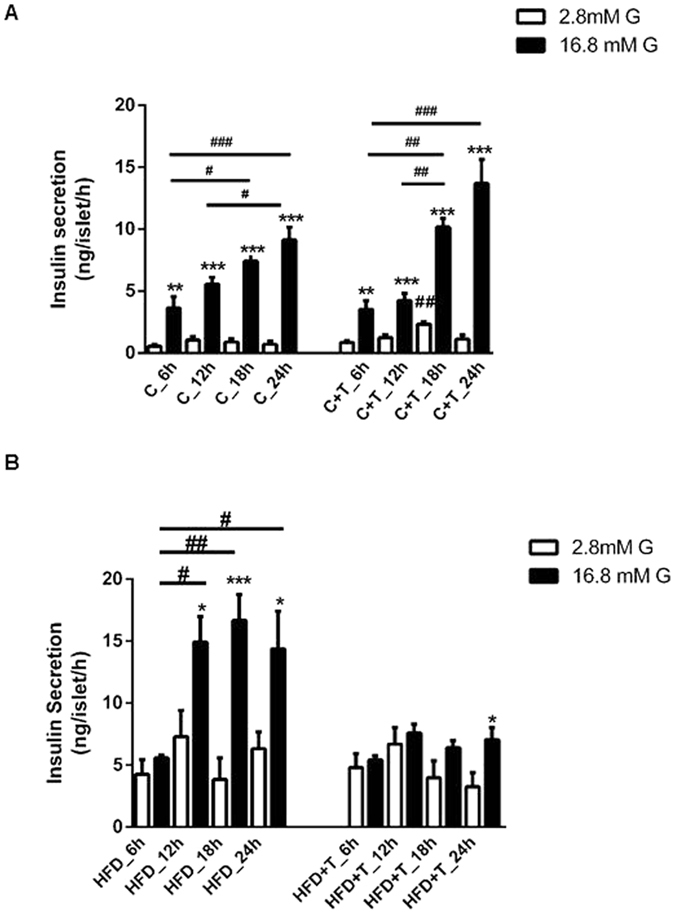
Effects of taurine on circadian insulin secretion *in vitro*. Pancreatic islets were isolated at different times of day (6:00 h, 12:00 h, 18:00 h and 24:00 h) after 10 weeks of taurine treatment and stimulated with 2.8 mM glucose (white bars) and 16.8 mM glucose (black bars) (n = 6–7 mice per group). (**A**) Insulin secretion from C and C+T groups. Data are expressed as mean ± SEM. Differences between 2.8 mM glucose and 16.8 mM glucose in the C (**P < 0.01, ***P < 0.001) and C+T groups (**P < 0.01, ***P < 0.001). Differences in the time of day at 2.8 mM glucose and 16.8 mM glucose in the C and C+T groups (^#^P < 0.05, ^##^P < 0.01, and ^###^P < 0.001 respectively). (**B**) Insulin secretion from HFD and HFD+T groups (n = 6–7). Differences between 2.8 mM glucose and 16.8 mM glucose in the HFD group (*P < 0.05, ***P < 0.001) and HFD+T group (*P < 0.05). Differences in the time of day at 16.8 mM glucose in HFD group (^#^P < 0.05, ^###^P < 0.001).

To check whether the modulation of taurine on the 24 h pattern of insulin secretion *in vivo* reflects changes in insulin secretion *in vitro*, we next performed glucose-stimulated insulin secretion from fresh isolated islets at 6:00, 12:00, 18:00, and 24:00. Isolated islets from control mice exhibited no changes in insulin secretion during the 24 h period at low glucose concentrations. Islets from the C+T group had increased insulin secretion at 18:00 when stimulated with basal glucose concentrations (Fig. 4A). When control islets were stimulated with high glucose concentrations, we could observe the effect of the time of the day on insulin secretion. Glucose-stimulated insulin secretion had a peak of secretion at 18:00 and maintained high levels at 24:00 as compared to secretion at 6:00 and 12:00. The same pattern of glucose-stimulated insulin secretion was observed in islets from the C+T group, however, taurine treatment augmented glucose-stimulated insulin secretion during the dark cycle, as compared to secretion from control islets (Fig. 4A). When islets from HFD mice were stimulated with high glucose concentrations, we found alterations in insulin secretion according to the time of day. Islets isolated at 6:00 lost the stimulatory effect of glucose compared to control islets (Fig. 4B). The nocturnal increase of glucose-stimulated insulin secretion was advanced to 12:00, exhibiting an increase in insulin secretion already at 12:00 and keeping high levels throughout the dark cycle. We could not detect the effect of the time of day in isolated islets from mice treated with HFD+T on glucose-stimulated insulin secretion. However, islets from HFD+T decreased glucose-induced insulin secretion at 12:00, 18:00 and 24:00, as compared to the HFD group (Fig. 4B). These results indicate that HFD disrupts *in vitro* glucose-stimulated insulin secretion that could not be prevented by taurine treatment.

### Effects of taurine on the clock gene expression in pancreatic islets

**Figure 5:**
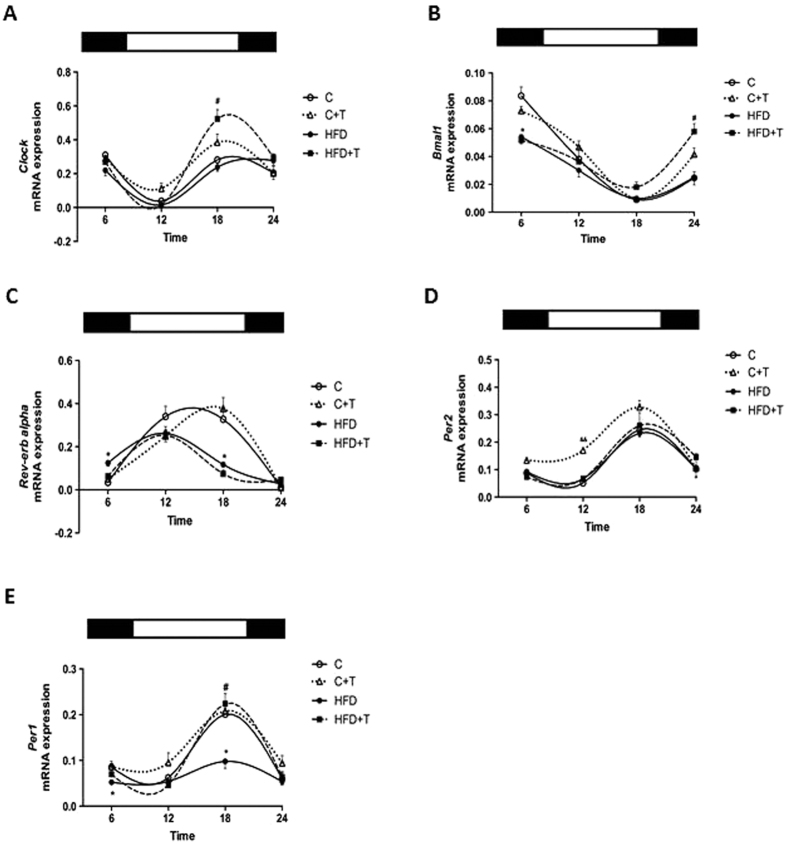
Clock genes expression in islets during 24 h. Pancreatic islets were isolated at different times of day (6:00 h, 12:00 h, 18:00 h and 24:00 h) after 10 weeks of taurine treatment. (n = 4–5). (**A**) *Clock* gene expression in isolated islets. 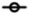 (C), 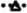 (C+T), 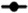 (HFD) and 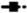 (HFD+T). (**B**) *Rev-erb alpha* gene expression in islets. (**C**) *Bmal1* gene expression in islets. (**D**) *Per2* gene expression in islets. (**E**) *Per1* gene expression in islets. Data are expressed as mean ± SEM. Differences between C vs C+T (^&&^P < 0.01), C vs HFD (*P < 0.05) and HFD vs HFD+T (^#^P < 0.05) (n = 6–7 mice per group). The black bars refer on the top of the figures to the dark cycle and the white bars to the light cycle.

The islet exhibits oscillations in clock gene expression throughout the day and alterations in clock gene expression impaired beta-cell function leading to the development of diabetes^8^,^9^,^10^,^19^. Therefore, we next checked whether taurine could modulate the daily pattern of *Bmal1*, *Clock*, *Rev-erbα*, *Per1* and *Per2* expression in isolated pancreatic islets. The expression of *Clock* was similar in control or C+T islets (Fig. 5A). There was no effect of HFD on *Clock* expression, but taurine treatment in the HFD group increased *Clock* expression at 18:00 (Fig. 5A). This effect was evident by the mesor and amplitude values showing an increase in the HFD+T group (Table 3). The 24 h pattern of *Bmal1* in control islets showed the highest peak at 6:00 and the lowest at 18:00 (Fig. 5B). Taurine treatment in mice fed with chow diet showed an increase in mesor values detected by the cosinor analysis. Islets from HFD downregulated *Bmal1* expression at 6:00, as compared to control islets, whereas HFD+T islets exhibited increased *Bmal1* expression at 24:00 (Fig. 5B). Cosinor analysis showed that HFD feeding decreased the mesor and amplitude of *Bmal1* expression and that taurine treatment restored mesor values of *Bmal1* expression and changed the acrophase (from 6 in all groups to 23 h) in mice treated with HFD (Table 3). *Rev-erbα* expression showed a similar pattern of expression in C and C+T islets, with a peak of expression at 12:00 and 18:00 and a decrease at 6:00 and 24:00 (Fig. 5C). HFD disrupted 24 h expression by upregulating *Rev-erbα* expression at 6:00 and downregulating at 18:00, compared to C islets, while there was no effect of taurine in HFD+T islets on *Rev-erbα* expression (Fig. 5C). Cosinor analysis confirmed this data, showing a decrease in the values of the mesor in the HFD group, in relation to the C group (Table 3). *Per2* expression had a peak of expression at 18:00 in control islets in all experimental groups, with increased *Per2* levels in the C+T group at 12:00 compared to control. There was no effect of diet and taurine among the other groups (Fig. 5D). In C and C+T islets, *Per1* showed changes during the 24 h pattern of expression with the highest peak at 18:00 (Fig. 5E). HFD downregulated *Per1* expression at 6:00 and at 18:00, as compared to C islets. Interestingly, taurine treatment during HFD feeding restored the circadian pattern of *Per1* expression to the same expression levels as in the C islets (Fig. 5E). Cosinor analysis confirmed the inhibitory effect of HFD on *Per1* expression and in the amplitude of the gene, as well as the preventive effect of taurine on the *Per 1* expression and *Per1* amplitude (Table 3). These results demonstrated that taurine treatment during HFD feeding can prevent the disruption of *Per1* expression in pancreatic islets.

### PER1 protein expression in pancreatic islets

**Figure 6:**
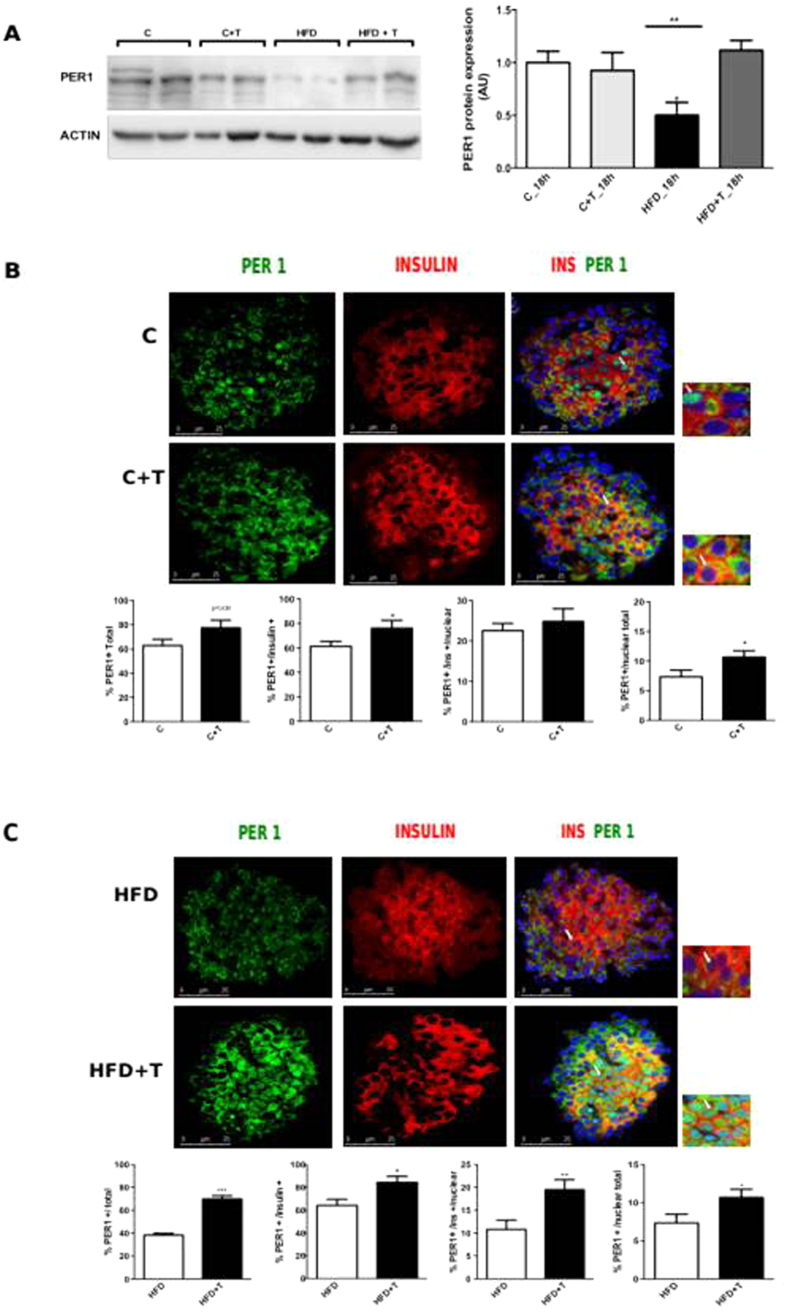
Effects of taurine in the expression of PER1 protein in isolated pancreatic islets. Pancreatic islets were isolated at 18:00 h after 10 weeks of taurine treatment in all experimental groups. Protein expression was detected by western blot and immunofluorescence stained against insulin anti-body (red), PER1 anti-body (green) and nuclear fraction by DAPI (blue). (**A**) PER1 protein expression normalized by actin. (n = 4–5 mice per group). (**B**) Percentage of PER1 expression in total number of cells, percentage of PER1 expression in the cytoplasm and the nucleus of insulin-positive cells, percentage of nuclear PER1 in beta-cells and percentage of nuclear PER1 in the total number of cells in chow diet groups. Differences between C (white bars) versus C+T (black bars). Data are expressed as mean ± SEM. Differences between C vs C+T (*P < 0.05) (n = 15–20 islets analyzed per condition per group). (**C**) Percentage of PER1 expression in total number of cells, percentage of PER1 expression in the cytoplasm and the nucleus of insulin-positive cells, percentage of nuclear PER1 in beta-cells and percentage of nuclear PER1 in the total number of cells in high fat diet groups. Differences between HFD (white bars) versus HFD+T (black bars). Data are expressed as mean ± SEM. Differences between HFD vs HFD+T (*P < 0.05, **P < 0.01, ***P < 0.001, n = 15–20 islets analyzed per condition per group).

Since taurine modulated the expression of *Per1* in islets, we next quantified protein levels of PER1 in pancreatic islets isolated at 18:00 and 24:00 after 10 weeks of treatment. Confirming our results, the HFD decreased the protein expression of PER1 at 18:00, and taurine restored PER1 protein levels in islets from mice treated with a HFD (Fig. 6A). As expected, there was no difference in PER1 protein expression at 24:00, confirming our results on gene expression (P = 0.46).

To further validate these results, immunofluorescence was performed in whole fresh isolated pancreatic islets of mice after 10 weeks of treatment. We analyzed total percentage of PER1-positive stained cells within the islet and found a higher tendency for PER1 staining in C+T islets (P = 0.09), as compared to control islets (Fig. 6B). Moreover, C+T islets showed a statistically significant increase in the percentage of total PER1 in insulin-positive cells when compared with C islets (Fig. 6B). Since PER1 is a transcription factor that can be localized in the cytoplasm and/or nucleus, we accessed the cell localization of PER1 in isolated islets. We found similar nuclear PER1 localization in the beta-cells of the C and C+T groups (Fig. 6B). However, when measuring nuclear staining in all islet cells, PER1 expression was upregulated by taurine in the control group (Fig. 6B).

Consistent with our results on gene expression, the HFD downregulated PER 1 protein expression as compared to C islets (P = 0.002). When we compared islets from HFD and HFD+T, we found an increase in total PER1-positive staining with taurine (Fig. 6C). Moreover, insulin-positive cells from HFD+T islets presented higher total PER1 labelling than islets from HFD mice (Fig. 6C). We observed an increased staining of nuclear PER1 from HFD+T islets and nuclear PER1 staining when analyzed in total islet cells, compared to islets from HDF mice (Fig. 6C). Thus, these results demonstrated that HFD downregulated PER1 protein and gene expression, whereas taurine upregulated PER1 in mice fed a HFD.

### Effects of taurine on the clock gene expression on visceral adipose tissue

We next checked whether taurine could modulate the daily pattern of *Bmal1*, *Clock*, *Rev-erbα*, *Per1* and *Per2* expression in visceral adipose tissue. The expression of *Clock* was similar in control or C+T islets (Supplementary Fig. 3A). However, HFD disrupted *Clock* expression in visceral adipose tissue by increasing *Clock* expression at 6:00 and at 18 and 24:00. Taurine treatment was able to restore the *Clock* expression at 18:00 and 24:00 in mice fed with HFD (Supplementary Fig. 3A). *Bmal1* expression in control group had a peak of expression at 6:00 and 24:00, and was decreased in C+T group at 6:00 and 24:00. *Bmal1* expression was downregulated by HFD at 12:00 and 24:00 but taurine treatment had no effect on *Bmal1* expression (Supplementary Fig. 3B). The expression of *Rev-erbα* in visceral adipose tissue was decreased at 6:00 and increased at 24:00 by HFD (Supplementary Fig. 3C) and taurine treatment could normalize *Rev-erbα* mRNA levels at 6:00 and 24:00. *Per1* expression was also decreased at 6:00 in visceral adipose tissue from mice fed a HFD but in this case, taurine treatment had no effect on *Per1* expression in visceral adipose tissue (Supplementary Fig. 3D). *Per 2* expression was increased in C+T group at 24:00 compared to control. In addition, HFD disrupted *Per2* expression by upregulating the expression of this gene at 6:00 and 24:00 whereas taurine treatment restored *Per2* expression at both 6 and 24:00 in mice treated with HFD (Supplementary Fig. 3E). Thus, taurine treatment during HFD feeding can prevent the disruption of *Clock*, *Rev-erbα* and *Per2* expression in visceral adipose tissue.

## Discussion

Close ties are found among nutrients, metabolic diseases and circadian clocks. This paper is the first to study the modulation of circadian rhythms by taurine. Taurine is one of the most abundant sulphonic acids in all tissues, and the efficacy of taurine administration against obesity and type 2 diabetes has been well documented. However, the impact of taurine on circadian clocks has not been studied before. In our results, HFD treatment significantly increased body weight and visceral fat and decreased glucose tolerance and insulin sensitivity, as previously established in other studies^10^,^20^,^21^. Taurine treatment prevents increases in body weight, glucose intolerance, insulin resistance and reduced visceral fat, according to studies where taurine had several beneficial metabolic effects in different models of type 2 diabetes and obesity^22^,^23^,^24^,^25^,^26^.

It has been demonstrated that consumption of a HFD disrupts the 24 h pattern of circulating levels of several hormones and the behavioural and molecular circadian rhythms in rodents^10^,^27^,^28^. We found that HFD increments food intake already at the first week of treatment during the light and dark cycles and that taurine treatment prevents HFD-increased food intake during both resting and active periods at the first and at 10^th^ week of treatment. Since the effect on food intake occurs already at the first week of treatment before any changes in body weight and fat weight this, suggest an early a central effect of taurine on food intake. Indeed, this effect could be explained by a direct effect of taurine on the hypothalamic regions that control food intake. Taurine has an anorexigenic action in the hypothalamus and modulates hypothalamic neuropeptide expression^29^,^30^. Another possibility could be a direct effect of taurine on the secretion of hormones that regulate food intake, including insulin and leptin. Indeed, we demonstrate that taurine treatment in mice fed with a HFD decreased the overall levels of plasma insulin during 24 h; it also decreased the insulin levels during the glucose tolerance test and diminished glucose stimulated insulin secretion in isolated islets. The 24 h pattern of plasma leptin levels was also reduced in mice treated with HFD+T, which could be explained by the decrease in visceral fat in these animals.

Diurnal variations in glucose, leptin and plasma insulin levels are subjected to daily cycles, which are altered in diabetes and obesity^10^,^31^,^32^,^33^,^34^,^35^,^36^,^37^. Our results confirm the disruption of leptin levels by HFD feeding and provide new insights regarding the preventive effect of taurine on leptin levels in animals fed a HFD. The mechanisms underlying the preventive effects of taurine on leptin levels are not yet known, but it is possible that taurine, by regulating insulin levels, could normalize the 24 h pattern leptin levels. In fact, daily leptin levels were shown to be dependent on insulin levels in mice^38^.

The daily pattern of insulin was shown to be independent of the temporal distribution of feeding in rats^34^, and an intrinsic circadian oscillator composed of clock and clock-controlled genes was found in mice, rats and humans^8^,^9^,^10^,^39^,^40^. Although we could not detect a 24 h pattern of insulin levels in our *in vivo* results, glucose-stimulated insulin secretion in isolated islets changed according to the time of day. Insulin secretion in control islets stimulated with glucose were elevated during the active period and reduced during the resting period, with enhanced circadian insulin secretion in mice fed a chow diet and treated with taurine. The 24 h pattern of glucose-stimulated insulin secretion was altered in islets from HFD mice, which showed a peak in insulin secretion during the resting period, consistent with the increase in food intake during the light cycle in these mice. Surprisingly, taurine treatment abolished the daily pattern of glucose-stimulated insulin secretion in isolated islets from HFD-fed mice. The reason is not known, but it could be possible that taurine could modulate important metabolic genes involved in the regulation of insulin secretion and beta-cell mass, as shown in previous studies with taurine supplementation in obese animals^41^,^42^.

The islet clock exhibits oscillations in clock gene expression throughout the day in different species, and alterations in their expression leads to impairments in insulin secretion^8^,^10^,^13^,^43^,^44^. Our results show that the HFD disrupts the expression of *Bmal1*, *Rev-erb alpha* and *Per1* genes in isolated islets, whereas taurine supplementation could not rescue *Bmal1* and *Rev-erbα* expression. An interesting finding of the present study is that taurine could prevent the downregulation of *Per1* levels caused by a HFD in isolated islets. This was evident in measurements of gene and protein expression, mainly in the insulin-positive cells. The increase in percentage of PER1 expression in insulin-positive cells was detected in the nuclear fraction of these cells, suggesting that PER1 could be a target of taurine. The role of PER1 in the regulation of insulin secretion has never been demonstrated, but it could be possible that taurine modulation of PER1 expression could regulate insulin secretion.

Adipose tissue clocks have important role in maintenance of lipid homeostasis and are related with features of obesity. The expression of clock genes in visceral adipose tissue were altered during HFD feeding. Similar peaks according to the diurnal expression of *Bmal1*, *Rev-erbα* and *Per 1*, *Per 2* during HFD were comparable with previous works respectively^28^,^45^. Taurine changed the expression of *Clock*, *Rev-erbα* and *Per2*, these effects of taurine could be tissue-dependent and peripheral clocks may be possible targets of this sulfonic acid during obesity.

Considering the different effects of *in vivo* taurine treatment on whole body metabolism, it is difficult that one mechanism could explain the taurine effects found in this study. However, it is clear from the present study that taurine improves disturbances in the 24 h pattern of plasma insulin and leptin, as well as *Per1* expression in pancreatic islets caused by HFD feeding. Thus, this is the first evidence that shows that taurine could be a potential target to correct or ameliorate the disturbances in circadian rhythms caused by obesity.

## Materials and Methods

### Animals and experimental groups

Ten-week-old male C57BL/6 mice were fed ad libitum with chow diet or high fat diet (45% fat Research diets Inc. D12451) for 10 weeks. The experimental groups were divided: Controls fed with chow diet (C), controls fed with chow diet and 2% taurine (Sigma-Aldrich, St. Louis, MO) in drinking water (C+T), mice fed with HFD (HFD) and mice fed with HFD and 2% taurine in water (HFD+T). Mice were allowed free access to food and water and maintained a 12 h light–dark cycle at 24 °C and constant humidity in soundproof cages. Body weight and water intake were measured every week during the 10 weeks of diet. Food intake was monitored from the first week of treatment by measuring the amount of food consumed from 8:00 to 20:00 (light cycle) and from 20:00 to 8:00 (dark cycle). After 10 weeks of treatment, mice were euthanized at different times of day: 6:00, 12:00, 18:00 and 24:00 h. Protocols were approved by the Animal Research Committee of the University of Barcelona, Spain, and principles of laboratory animal care were followed, according to European and local government guidelines.

### Daily measurements of glucose, insulin and leptin

The measurements of daily pattern of glucose, insulin and leptin were done in the fed state at the end of the long-term treatment of 10 weeks. Animals were sacrificed at the following times 6:00, 12:00, 18:00 and 24:00 and blood samples were collected to measure plasma insulin and leptin by ELISA (Mercodia Insulin, Uppsala Sweden and Crystal Chem, Harris County, TX, USA, respectively). Blood glucose was measured with glucometer (Accu-Check; Roche Diagnostics, Madrid, Spain).

### Glucose and insulin tolerance tests

Glucose tolerance and insulin sensitivity were assessed during the last week of treatment. In the intraperitoneal glucose tolerance tests, 4 h-fasted mice were injected with 2 g glucose/kg body weight; blood glucose levels were then measured at 0, 15, 30, 60, 90 and 120 min after injection, and glucose was measured at all points using a glucometer (Accu-Check; Roche Diagnostics, Madrid, Spain). Moreover, blood samples were collected at the first 4 time points to quantify insulin. For insulin tolerance tests, mice were injected with 0.50 IU insulin/kg body weight. Blood samples were collected before insulin injection (time 0) and at 15, 30, 60, 90 min and 120 min after insulin administration, for glucose measurements. Plasma was separated by centrifugation. Plasma levels of insulin and leptin were measured by ELISA (Mercodia Insulin, Uppsala Sweden, and Crystal Chem, Harris County, TX, USA, respectively).

### Isolation of pancreatic islets

After being fully anesthetized, mice were euthanized by cervical dislocation and a collagenase P solution (Sigma-Aldrich, St. Louis, MO, USA) was perfused into the pancreas through a direct puncture of the common bile duct to digest the pancreas. After digestion, islets of Langerhans were separated from exocrine tissue using a density gradient with Histopaque (Sigma-Aldrich, St. Louis, MO, USA). Islets were handpicked in a Leica stereomicroscope for further measurements of gene and protein expression and for insulin secretion assays.

### Glucose-stimulated insulin secretion *in vitro*

After 10 weeks of treatment, mice were euthanized at 6:00, 12:00, 18:00 and 24:00. Groups of 5 fresh isolated islets from 4–5 different mice from each group were first pre-incubated at 37 °C in a 5.6 mM glucose KRBH solution for 30 min. Supernatant was discarded, and islets were incubated for 60 min at 37 °C in KRBH containing 2.8 mM or 16.7 mM glucose, respectively. After incubation, supernatants were collected, and insulin was quantified using a mouse insulin ELISA kit (Mercodia Insulin Uppsala, Sweden).

### Western Blot

Protein extraction of isolated islets was obtained using RIPA lysis buffer (Tris 50 mmol/l, pH 7.5, EDTA 5 mmol/l, NaCl, 1% 150 mmol/l, Triton X-100 1%, SDS 0.1%, 10 mmol/l sodium fluoride, 1% sodium deoxycholate, and protease inhibitors). For the extraction, islet lysates were frozen and thawed twice after centrifugation for 20 min at 4 °C, and supernatants were collected and stored at −80 °C. Protein quantification was determined with the Lowry protein assay kit (Bio-rad, Hercules, CA, USA). The protein was electrotransferred onto a PVDF membrane. The membranes were blocked for 1 h with 0.05% Tween-20 and 5% NFDM, and then incubated overnight at 4 °C with the antibodies: AntiPer1 (1:500; Thermo Scientific Inc.) ß-Actin was used as a loading control (1:5,000; GE Healthcare, Hertfordshire, UK). Protein bands were revealed by using the Pierce ECL western blot substrate (Thermo Fisher Scientific, Madrid, Spain). Respective bands were quantified by densitometry. Image J software 1.50a and intensity values for PER 1 were normalized with ß-Actin.

### RNA isolation and Real-time PCR

Total RNA was prepared from isolated islets using the RNeasy Mini Kit (Qiagen, Hilden, Germany). RNA was quantified using a Nanodrop 1000 (Thermo Scientific Wilmington, MA) and then retrotranscribed using the High Capacity cDNA Reverse Transcription Kit (AB Applied Biosystems, USA) following the manufacturer’s instructions. The cDNA was amplified by Real-Time PCR in a LightCycler 480 System (Roche) using Mesa Green qPCR Master Mix (Mesa Green, Eurogentec, Belgium). The expression of clock genes in isolated pancreatic islets was measured, with the housekeeping gene *36B4* (*ribosomal protein large P0*) used as the endogenous control for quantification. The results were expressed as the relative expression with respect to control levels (2^−ΔΔct^). The primer sequences are shown in Table 4.

### Whole islet immunofluorescence

Fresh isolated islets were washed in buffer with PBS 1x and Triton 0.2%, then fixed in 4% of Paraformaldehide (Electron Microscopy Sciences, Hatfield, PA, USA) for 20 min, permeabilized with PBS with Triton 0.3% and blocked in a PBS solution with Triton 0.5% and FBS 10% for 1 h, and incubated overnight with the following primary antibodies: guinea pig anti-insulin 1:500, (Dako, Glostrup, Denmark) and anti-Per1 1:100 (Thermo Scientific, CA). Islets were incubated 2 hours with Secondary antibodies Cy3-labeled anti-guinea pig (Jackson Immuno research) and Alexa Fluor 488 (anti-rabbit (Molecular Probes, USA) were used at 1:200 dilutions, respectively. Nuclear staining was performed by using mounting media with DAPI (Life Technologies, USA). Immunofluorescence was assessed in a confocal laser microscope (Leica. Microsystems, Wetzlar Germany). For each individual islet images were acquired every 10 μm using a 40x oil immersion objective.

The same settings (i.e pinhole, smart gain, smart offset, phase, zoom) were maintained for each islet in all groups. Images were analyzed by Image J Software 1.50a (NIH, USA). Analysis was done by counting the number of stained positive cells represented by percentage.

### Statistical analysis

Values are presented as means ± SEM. Differences between two groups were analyzed by Student’s *t* test. The effect of time and groups differences were measured by one-way or two-way ANOVA, with Bonferroni post hoc test for multiple comparisons, using GraphPad Prism software where appropriate. All statistical tests were performed with a level of significance P < 0.05. Cosinor Analysis was done using the Acrophase program (R. Refinetti 2004) for fitting cosine functions to the data using a fixed 24 hour period and included the mesor (middle value of the fitted cosine representing a rhythm-adjusted mean), the amplitude (difference between the minimum and maximum of the fitted cosine function), the acrophase (the time at which the peak of a rhythm occurs, expressed in hours) and fitted cosine values to calculate the goodness of the fit by coefficient of determination R^2^.

## Additional Information

**How to cite this article**: Figueroa, A. L. C. *et al*. Taurine Treatment Modulates Circadian Rhythms in Mice Fed A High Fat Diet. *Sci. Rep*. **6**, 36801; doi: 10.1038/srep36801 (2016).

**Publisher’s note:** Springer Nature remains neutral with regard to jurisdictional claims in published maps and institutional affiliations.

## Supplementary Material

Supplementary Information

## Figures and Tables

**Table 1:** Effects of taurine treatment on body weight, visceral fat and food intake.

Variable	Groups				p Value interactions		
	C	C+T	HFD	HFD+T	C vs C+T	C vs HFD	HFD vs HFD+T
Body weight gain (g)	4 ± 0,8	3,7 ± 0,5	14,5 ± 0,9^***^	8,7 ± 0,8^##^	0,76	0,001	0,01
Visceral fat (mg)	596,4 ± 109,7	673,5 ± 112,6	1866,3± 183,5^***^	1229,8 ± 214,7^#^	0,63	0,001	0,03
Final Food intake (kcal/day/mouse)	6,4 ± 0,3	5,9 ± 0,3	11,05 ± 0,4^***^	8,5 ± 0,4^#^	0,32	0,001	0,05
Differences between groups							
C vs C+T ns (not significant)							
C vs HFD	^***^p < 0.05						
HFD vs HFD+T	^#^p < 0.05						
	^##^p < 0.01						

Effect of HFD and taurine after 10 weeks of treatment. Values shown are the means ± SEM. (Two-way ANOVA followed by Bonferroni post hoc test).

**Table 2:** Cosinor analysis of the 24 h expression of metabolic parameters.

	R^2^				Mesor				p Value			Amplitude				p Value			Acrophase				p Value		
	C	C+T	HFD	HFD+T	C	C+T	HFD	HFD+T	C vs C+T	C vs HFD	HFD vs HFD-T	C	C+T	HFD	HFD+T	C vs C+T	C vs HFD	HFD vs HFD-T	C	C+T	HFD	HFD+T	C vs C+T	C vs HFD	HFD vs HFD-T
Glucose	0,028	0,02	0,001	0,003	123,7	116^&&^	136,5^**^	121,17^###^	0,01	0,005	0,001	11,6	12,9	12,1	9,4	0,76	0.85	0.25	11,0	23,0^&&&^	17,0	17,0	0,001	0,96	0,9
Insulin	0,24	0,38	0,01	0,60	1,4	1,5	9,3^***^	2,9^##^	0,87	0,001	0,007	0,6	0,7	3,3^***^	1,8^#^	0,7	0.001	0.04	23,0	23,0	17,0	23,0	0,77	0,35	0,9
Leptin	0,49	0,34	0,06	0,48	3,5	4,6	64,3^***^	38,9^##^	0,11	0,001	0,007	2,6	2,5	27,4^***^	15,8^##^	0,61	0.001	0.01	23,0	23,0	17,0	23,0	0,32	0,68	0,2
Differences between groups																									
C vs C+T	^&&^p < 0.01																								
	^&&&^p < 0.001																								
C vs HFD	**p < 0.01																								
	***p < 0.01																								
HFD vs HFD+ T	#p < 0.05																								
	##p < 0.01																								
	###p < 0.001																								

Cosinor analysis including goodness of the fit (**R**^2^), mesor, amplitude, and acrophase of the 24 h profiles of plasma glucose, insulin and leptin. Next Two-way ANOVA shows the difference between groups in mesor, amplitude and acrophase respectively.

Values shown are the means ± SEM.

**Table 3:** Cosinor analysis of the 24 h expression of clock genes.

	R^2^				Mesor				p Value			Amplitude				p Value			Acrophase				p Value	
	C	C+T	HFD	HFD+T	C	C+T	HFD	HFD+T	C vs C+T	C vs HFD	HFD vs HFD+T	C	C+T	HFD	HFD+T	C vs C+T	C vs HFD	HFD vs HFD+T	C	C+T	HFD	HFD+T	C vs C+T	C vs HFD
*Rev-erb α*	0,74	0,82	0,43	0,42	0,17	0,17	0,11^*^	0,08	0,91	0,05	0,25	0,17	0,18	0,11	0,09	0,67	0.17	0.55	11,0	17,0^&^	11,0	11,0	0,05	0,34
*Clock*	0,23	0,08	0,46	0,30	0,17	0,24	0,18	0,26^#^	0,13	0,63	0,05	0,16	0,23	0,16	0,28^#^	0,21	0.89	0.05	23,0	18,0	23,0	18,0	0,91	0,87
*Bmal1*	0,80	0,65	0,39	0,54	0,04	0,47^&&&^	0,03^**^	0,04^#^	0,001	0,01	0,02	0,04	0,04	0,02^*^	0,03	0,99	0.02	0.74	6,0	6,0	6,0	23,0^&^	0,57	0,22
*Per1*	0,46	0,42	0,36	0,60	0,10	0,12	0,06^*^	0,10^#^	0,12	0,05	0,05	0,07	0,09	0,03^*^	0,09^##^	0,20	0,04	0,01	17,0	17,0	18,0	18,0	0,40	0,42
*Per2*	0,41	0,37	0,46	0,36	0,12	0,20^&&^	0,12	0,12	0,01	0,85	0,90	0,09	0,13	0,08	0,10	0,31	0,50	0,63	17,0	17,0	19,0	19,0	0,20	0,34
Differences between groups.																								
C vs C+T	^&^p < 0.05																							
	^&&^p < 0.01																							
	^&&&^p < 0.01																							
C vs HFD	*p < 0.05																							
	**p < 0.01																							
HFD vs HFD+ T	#p < 0.05																							
	##p < 0.01																							

Cosinor analysis including goodness of the fit **(R**^**2**^), mesor, amplitude, and acrophase of the 24 h profiles of clock genes in pancreatic islets. Next Two-way ANOVA shows the difference between groups in mesor, amplitude and acrophase respectively.

Values shown are the means ± SEM.

**Table 4:** Quantitative real PCR primers.

Name	Sense primer (5′3)	Antisense primer (5′3)
*Rev-erbα*	GGTGCGCTTTGCATCTT	GGTTGTGCGGCTCAGGAA
*Clock*	TTGCTCCACGGGAATCCTT	GGAGGGAAAGTGCTCTGTTGTAG
*Bmal1*	GGACTTCGCCTCTACCTGTTCA	AACCATGTGCGAGTGCAGGCGC
*Per1*	GCGGGTCTTCGGTTAAGGTT	GCTCAGCTGGGATTTGG
*Per2*	ATGCTCGCCATCCACAAGA	GCGGAATCGAATGGGAGAAT
*36b4*	GAGGAATCAGATGAGGATATGGGA	AAGCAGGCTGACTTGGTTGC
