# Supplementary material for: Taurine Treatment Modulates Circadian Rhythms in Mice Fed A High Fat Diet
Source: Sci Rep. 2016 Nov 18;6:36801. doi: 10.1038/srep36801 (PMC5114685; doi:10.1038/srep36801)
Supplement: Supplementary Information [file srep36801-s1.pdf]

TAURINE TREATMENT MODULATES CIRCADIAN RHYTHMS IN MICE FED A HIGH FAT DIET

Ana Lucia C. Figueroa[1], Hugo Figueiredo[1], Sandra A. Rebuffat[1,2], Elaine Vieira[*2,3] Ramon Gomis[*1,2,4]

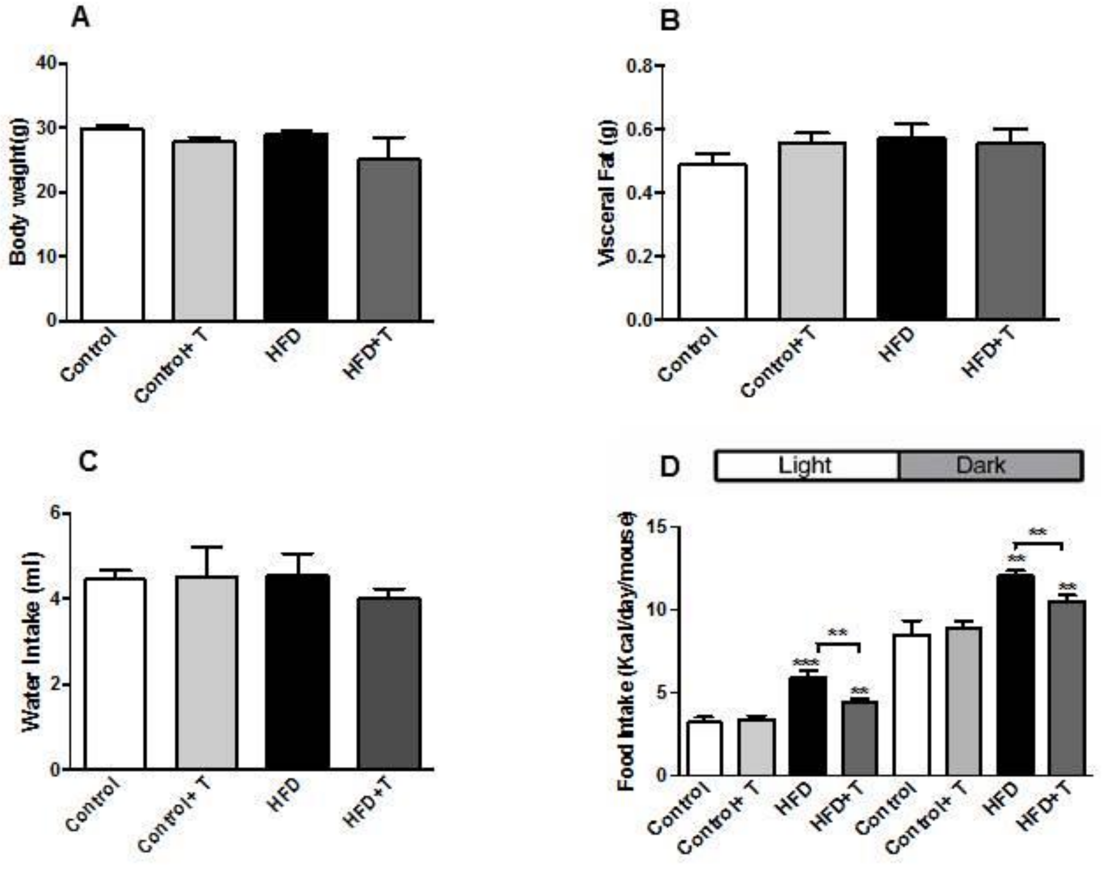

Supplementary Figure 1. Effects of taurine treatment on body weight, visceral fat, water intake and food intake after 1 week of treatment . (A) Body weight, (B) visceral fat, (C) water intake and (D) food intake during the light and dark cycles. (n=5-7 mice per group). Data are expressed as mean ± SEM. ** $p<0.01$ *** $p<0.001$

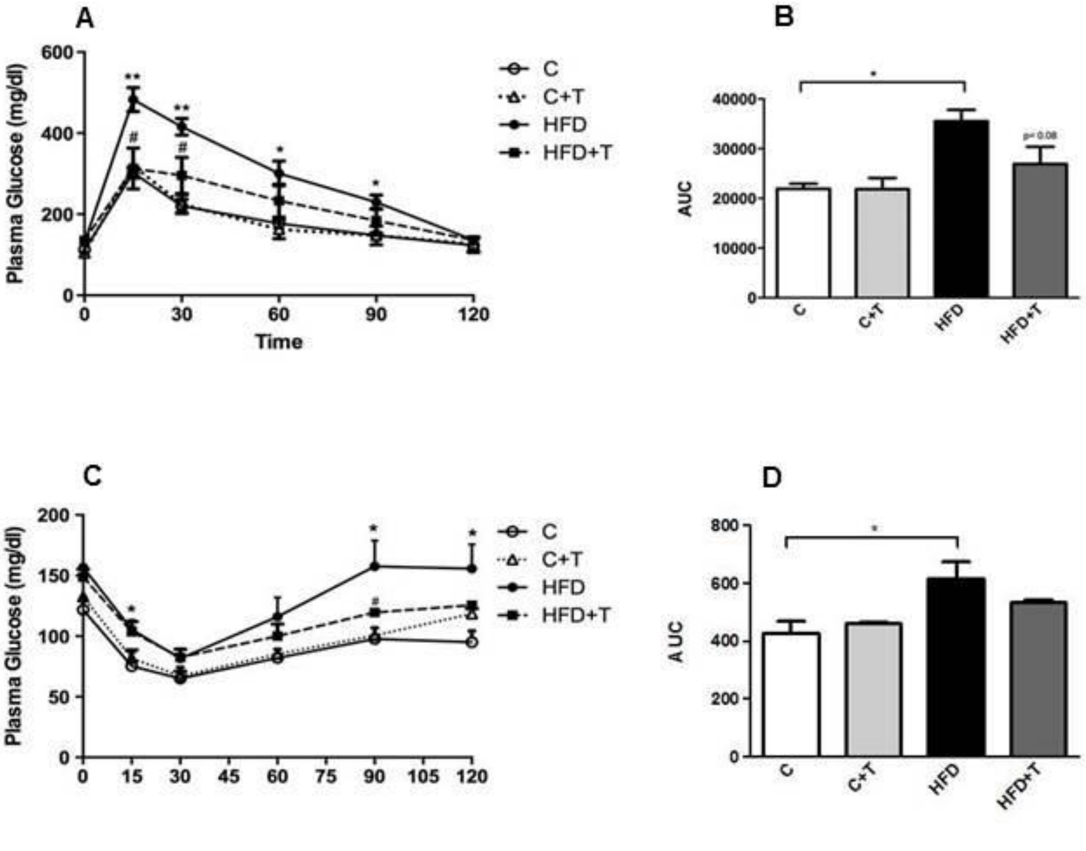

Supplementary Figure 2. Effects of taurine treatment on glucose tolerance and insulin sensitivity after 1 week of treatment. Glucose tolerance test **(A)**. Differences between C vs HFD (* $P < 0.05$, ** $P < 0.01$); HFD vs HFD+ T (# $P < 0.05$). (n=5-7 mice per group) **(B)** Area under the curve (AUC). **(C)** Insulin tolerance test. Differences between C vs HFD (* $P < 0.05$); HFD vs HFD+ T (# $P < 0.05$). (n=5-7 mice per group) **(D)** Area under the curve (AUC). Data are expressed as mean ± SEM.

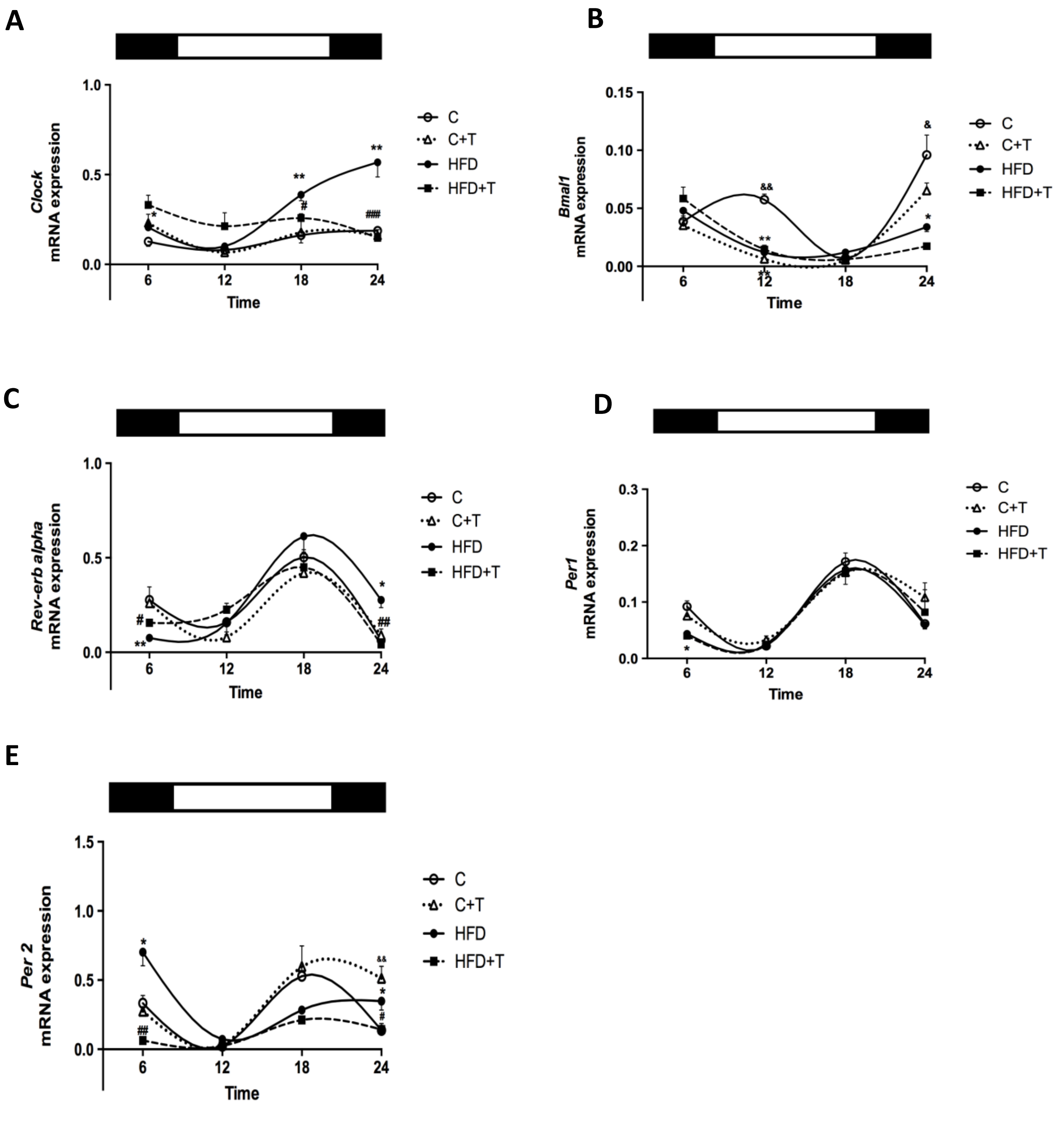

Supplementary Figure 3. Clock genes expression in adipose tissue during 24h.
Expression of clock genes in visceral adipose tissue at different times of day (6:00h, 12:00h, 18:00h and 24:00h) after 10 weeks of taurine treatment. (n=4-5). **(A)** *Clock* gene expression in adipose tissue (C), (C+T), (HFD) and (HFD+T). **(B)** *Rev-erb alpha* gene expression in adipose tissue. **(C)** *Bmal1* gene expression in islets. **(D)** *Per1* gene expression in adipose tissue. **(E)** *Per2* gene expression in adipose tissue. Data are expressed as mean ± SEM. Differences between C vs C+T ($^{\&}$ P<0.05, $^{\&\&}$ P<0.01), C vs HFD (* P<0.05, **P<0.01) and HFD vs HFD+T ($^{\#}$ P<0.05, $^{\#\#}$P<0.01, $^{\#\#\#}$P<0.001) (n=6-7 mice per group). The black bars refer on the top of the figures to the dark cycle and the white bars to the light cycle.
